# The Pituitary Adenylate Cyclase-Activating Polypeptide (PACAP) System of the Central Amygdala Mediates the Detrimental Effects of Chronic Social Defeat Stress in Rats

**DOI:** 10.1523/ENEURO.0260-22.2022

**Published:** 2022-09-15

**Authors:** Mariel P. Seiglie, Lauren Lepeak, Clara Velázquez-Sanchez, Antonio Ferragud, Teresa Le, Pietro Cottone, Valentina Sabino

**Affiliations:** Laboratory of Addictive Disorders, Departments of Pharmacology and Psychiatry, Boston University School of Medicine, Boston, MA 02118

**Keywords:** amygdala, anxiety, HPA, neuropeptide, PACAP, stress

## Abstract

Many psychiatric diseases stem from an inability to cope with stressful events, as chronic stressors can precipitate or exacerbate psychopathologies. The neurobiological mechanisms underlying the response to chronic stress and the resulting anxiety states remain poorly understood. Stress neuropeptides in the extended amygdala circuitry mediate the behavioral response to stress, and hyperactivity of these systems has been hypothesized to be responsible for the emergence of persistent negative outcomes and for the pathogenesis of anxiety-related and trauma-related disorders. Pituitary adenylate cyclase-activating polypeptide (PACAP) and its receptor PAC1R are highly expressed within the central amygdala (CeA) and play a key role in stress regulation. Here, we used chronic social defeat stress (CSDS), a clinically relevant model of psychosocial stress that produces robust maladaptive behaviors in rodents. We found that 10 days of CSDS cause a significant increase in PACAP levels selectively in the CeA of rats, as well as an increase in PAC1R mRNA. Using a viral vector strategy, we found that PAC1R knock-down in the CeA attenuates the CSDS-induced body weight loss and prevents the CSDS-induced increase in anxiety-like behavior. Notably, CSDS animals display reduced basal corticosterone (CORT) levels and PAC1R knock-down in CeA further reduce them. Finally, the CeA PAC1R knock-down blocks the increase in corticotropin-releasing factor (CRF) immunoreactivity induced by CSDS in CeA. Our findings support the notion that the persistent activation of the PACAP-PAC1R system in the CeA mediates the behavioral outcomes of chronic psychosocial stress independently of the hypothalamic-pituitary-adrenal axis, perhaps via the recruitment of the CRF system.

## Significance Statement

Our results identify a key role for the neuropeptide pituitary adenylate cyclase-activating polypeptide (PACAP) specifically of the central amygdala (CeA) in mediating the negative physiological and behavioral consequences of chronic stress, independently of the hypothalamus-pituitary-adrenal axis. This system may, therefore, represent a novel target for the treatment of stress-related psychopathologies such as anxiety-related disorders and post-traumatic stress disorder (PTSD).

## Introduction

Mental disorders are an enormous global health issue because of their high cost to society and their prevalence. In particular, in the United States anxiety disorders represent the most common mental illness, with a lifetime prevalence above 30% (Baxter et al., 2013; Roehrig, 2016; National Institute of Mental Health, 2021; Collaborators GBDMD, 2022), while the lifetime prevalence of posttraumatic stress disorder (PTSD) is 6.1–9.2% (Goldstein et al., 2016; Koenen et al., 2017). Chronic and traumatic stressors play a large role in the development of psychiatric diseases, as they can precipitate or exacerbate psychopathologies (de Kloet et al., 2005; Juster et al., 2010; Russo et al., 2012). Despite the ongoing and worsening mental health crisis, the neurobiological mechanisms underlying the pathologic response to chronic stress remain poorly understood. Multiple neurobiological systems are involved in the response to stress. The brain responds to states of threatened homeostasis by activating adaptive responses intended to maintain the equilibrium and prepare us for immediate or potential harm (Chrousos and Gold, 1992; Tovote et al., 2015). However, when stressors become repeated or chronic, hyperactivation of specific neurotransmitter systems may result in the emergence of persistent negative outcomes (Shin and Liberzon, 2010; Duval et al., 2015; Sapolsky, 2015).

The central nucleus of the amygdala (CeA) and the bed nucleus of the stria terminalis (BNST), both part of the extended amygdala, orchestrate the emotional component of the behavioral response to stress (Alheid and Heimer, 1988; Koob and Le Moal, 2005). The CeA integrates sensory information from the environment and projects information to effector regions to trigger appropriate responses to threats (Davis, 1992; Davis and Shi, 2000; Zarrindast et al., 2008). While in a nonpathologic state the amygdala signaling is tapered appropriately to the severity of the present threat (Mathew et al., 2008), this region is instead hyperresponsive in anxiety disorders (Etkin and Wager, 2007; Etkin et al., 2009; Shin and Liberzon, 2010; Fox et al., 2015).

Pituitary adenylate cyclase-activating polypeptide (PACAP) has been proposed to be a master regulator of the stress response (Dore et al., 2013; Hammack and May, 2015; Varodayan et al., 2020; Boucher et al., 2021a). PACAP, a 38-amino acid peptide belonging to the secretin/glucagon/vasoactive intestinal polypeptide (VIP) superfamily, exerts its effects mainly via its cognate receptor PAC1 (PAC1R), which binds PACAP with an affinity of 1000-fold greater than VIP (Harmar et al., 1998; Vaudry et al., 2009). Dense PACAP-immunoreactive fibers of nonlocal origin are found in the capsular and lateral parts of the CeA (CeC, CeL) and in the latero-dorsal BNST (STLD; Piggins et al., 1996; Hannibal, 2002; Zhang et al., 2021), while PACAP mRNA is highly expressed in hypothalamic and brainstem nuclei (Hannibal et al., 1995; Joo et al., 2004).

In humans, a single nucleotide polymorphism in the PAC1R gene is associated with PTSD symptoms in women, a mutation which is also associated with increased amygdala activity in response to threatening faces (Ressler et al., 2011; Stevens et al., 2014). In rodents, central administration of PACAP into the ventricles, hypothalamus, and extended amygdala evokes a stress-like response (Agarwal et al., 2005; Hammack et al., 2009; Stroth et al., 2011; Dore et al., 2013; Roman et al., 2014; Seiglie et al., 2015; Meloni et al., 2019). Exposure to acute stressors increases PACAP levels in both CeA and BNST (Seiglie et al., 2019), and chronic variate stress recruits PACAP in the BNST (Hammack et al., 2009; Roman et al., 2014). PACAP knock-out mice display an anxiolytic profile and attenuated endocrine, molecular, and behavioral responses to chronic stress (Hashimoto et al., 2001; Stroth and Eiden, 2010; Gaszner et al., 2012; Lehmann et al., 2013; Kormos et al., 2016). Consistent with PACAP’s ability to mediate stress responses, PACAP-immunoreactive fibers are found in close proximity to corticotropin-releasing factor (CRF) neurons (Hannibal et al., 1995; Légrádi et al., 1998; Missig et al., 2014); PACAP acts as an upstream regulator of CRF and many PACAP behavioral effects are prevented by CRF receptor antagonism (Tsukiyama et al., 2011; Dore et al., 2013; Seiglie et al., 2015; Miles et al., 2019). In the CeA, PACAP increases GABA release via PAC1R via a presynaptic mechanism (Varodayan et al., 2020), an action that mimics that of CRF itself (Roberto et al., 2010; Varodayan et al., 2017).

The chronic social defeat stress (CSDS) is a clinically relevant, highly translational model of psychosocial stress based on chronic social subordination, which produces robust maladaptive behaviors (Krishnan et al., 2007; Russo et al., 2012; Hammels et al., 2015). Indeed, defeated animals show a wide range of anxiety-like and depressive-like behaviors, as well as physiological changes, including decreased body weight gain (Berton et al., 2006; Krishnan et al., 2007; Iñiguez et al., 2016). Notably, while whole body PACAP gene deletion in mice has been shown to have no significant effects on affective behaviors in nonstressed mice, it instead led to a robust behavioral protection in CSDS animals, suggesting that PACAP may mediate the detrimental effects of CSDS (Lehmann et al., 2013). However, where in the brain PACAP is mediating the detrimental effects of CSDS is currently unknown.

Here, we hypothesized that the CeA PACAP/PAC1R system is recruited by CSDS and that it contributes to the resulting physiological and behavioral outcomes. We first assessed the effects of CSDS on PACAP levels in the CeA and BNST and measured PAC1R expression in CeA. We then evaluated the functional role of PAC1R by knocking down this receptor in the CeA via an AAV-shRNA and assessing its effects on body weight, anxiety-like behaviors, plasma corticosterone (CORT) levels, and CeA CRF levels.

## Materials and Methods

### Subjects

The experimental animals (intruders) were male Sprague Dawley rats (Envigo) weighing 301–325 g on arrival. Rats were single-housed in 10 1/2” × 19” × 8” wire-topped, plastic cages on a 12-h reverse light cycle (lights off at 11 A.M.), in an AAALAC-approved humidity-controlled and temperature-controlled vivarium. Food (Envigo Teklad LM-4857012 diet) and water were available *ad libitum*. Male Long–Evans retired breeders, 400–600 g on arrival, were used as residents, and housed in 20” × 16” × 8 1/2” wire-topped, plastic cages with ovariectomized Long–Evans females. Experimental tests were conducted during the rats’ dark cycle. Three groups of rats were used in the experiments; group sizes were as follow: group 1: CSDS PACAP immunohistochemistry (IHC) experiment [total 20 rats, eight controls (Ctrls.) and 12 CSDS]: CeA, *N* = 6–11/group (17 rats); BNST/paraventricular nucleus of the hypothalamus (PVN), *N* = 8–12/group (20 rats); group 2: CSDS PACAP quantitative real-time PCR (qPCR) experiment: *N* = 6–11/group (total 17 rats); group 3: AAV-PAC1R KD experiment: body weight, *N* = 9–12/group (total 43 rats); light-dark test, *N* = 9–10/group (39 rats); plasma CORT, *N* = 9–12/group (44 rats); CRF IHC, *N* = 6–9/group (30 rats). Procedures adhered to the National Institutes of Health *Guide for the Care and Use of Laboratory Animals* and the Principles of Laboratory Animal Care and were approved by the Institutional Animal Care and Use Committee.

### Social defeat stress

The CSDS paradigm was modified from the resident-intruder model originally designed by Miczek and colleagues (Miczek, 1979; Tidey and Miczek, 1996). The CSDS sessions, which occurred once a day on consecutive days between 5 and 7 P.M., consisted of an intruder rat being placed into the home cage territory of an unfamiliar resident, which had previously been trained for high aggression (Fekete et al., 2009). Exposure lasted until the intruder submitted (i.e., assumed a submissive, supine position for >3 s) or, if submission did not occur, up to 5 min, in which case the intruder was moved to a second resident and the session restarted. Upon submission, the intruder was then placed inside a wire mesh enclosure (7 × 9 × 8.5 inches) inside the resident cage for the remainder of the 30-min session, which allowed auditory, olfactory, visual, and limited physical contact (mouth/nose) but prevented injuries. Ctrl. rats were picked up, handled, and returned to their home cage for 30 min.

### PACAP IHC

#### Experimental details

A set of Ctrl. and CSDS (10 d, CSDS) rats were anesthetized with isoflurane and then transcardially perfused as previously described (Iemolo et al., 2013), 24 h after the last (10th) CSDS session. Coronal 30-μm sections were cut on a cryostat, collected, and stored in cryoprotectant at −20°C. Every sixth section (180 μm apart) for CeA (bregma range: −2.0 to −3.0 mm) and every fourth section (120 μm apart) for BNST (bregma range: 0.24 to −0.24 mm) were selected and processed for IHC.

#### PACAP staining

PACAP IHC was performed as previously described (Seiglie et al., 2019; Ferragud et al., 2021). Free-floating sections were washed in Tris-buffered saline (TBS) after every incubation. Sections were incubated in 0.3% hydrogen peroxide for 10 min to block endogenous peroxidases. Sections were then blocked for 1 h in 3% normal goat serum, 0.4% Triton X-100 and then transferred into an anti-PACAP primary antibody (Peninsula Labs, 1:8000) in blocking solution for 24 h at 4°C. Sections were then incubated in secondary antibody (1: 500, biotinylated anti-rabbit, Vector Laboratories) in blocking solution for 2 h at room temperature and finally incubated in an avidin–biotin horseradish peroxidase ABC solution (Vector Laboratories) in blocking solution for 1 h. Sections were then processed using a diaminobenzidine substrate kit (Vector Laboratories) until reaction was complete and mounted onto slides and allowed to dry overnight. The following day, slides were dehydrated and coverslipped using DPX mountant (Electron Microscopy Sciences).

#### Quantification of PACAP staining

Using the Stereo Investigator software (MicroBrightField), 10× objective pictures of sections containing either the CeA or the BNST were taken using an Olympus BX-51 microscope equipped with a Retiga 2000R live video camera (QImaging), a three-axis MAC6000 XYZ motorized stage (Ludl Electronics), and a personal computer workstation. Chromogen PACAP pictures were taken in bright field under a preset exposure and gain, to standardize the images. For each image, area contours were drawn corresponding to CeC and CeL for CeA and to the latero-dorsal part of the BNST (STLD), where PACAP immunoreactivity is observed. Densitometry analysis was performed using ImageJ software (NIH); mean optical density of signal was calculated by subtracting the background signal and then by normalizing the value to the traced area.

### Brain punching and qPCR

Tissue PAC1R and CRF mRNA levels were determined as previously described (Cottone et al., 2009; Sabino et al., 2011; Dore et al., 2013). Rats were anesthetized with isoflurane and brains were quickly removed and coronally sliced in a brain matrix; 1-mm diameter bilateral punches containing the CeA were collected on an ice-cold stage. Total RNA was prepared from tissue using the RNeasy Lipid Mini kit (QIAGEN); total RNA was quantified by Nanodrop 1000 (Thermo Scientific) and then reverse transcribed with QuantiTect Reverse Transcription kit (QIAGEN), which includes a DNA removal step. For qPCR, Roche Light Cycler 480 Master-plus Sybr Green mix (Roche Applied Science) was used. Reactions (10 μl) were conducted in a 96-well plate Realplex2 machine (Eppendorf). The primers (0.5 μm final concentration, Sigma), synthetized with a standard desalting purification, were the following: PAC1R, CAT GGT CAT CTT GTG CCG CTT CC and GAC TGC TGT CCT GCT CGG CGT ACA (94°C 15 s; 70°C 8 s); CRF, TGC TCG GCT GTC CCC CAA CT and CTG CAG CAA CAC GCG GAA AAA (95°C 10 s; 59.2°C 5 s; 72°C 10 s); Cyclophilin A (CypA), TAT CTG CAC TGC CAA GAC TGA GTG and CTT CTT GCT GGT CTT GCC ATT CC (95°C 20 s; 58°C 15 s; 72°C 20 s). Standard curves were constructed using sequenced PCR products. Results were analyzed by second derivative methods and then normalized to CypA expression levels. Standards and samples were run in duplicate. Gene-specific amplification was determined by melting curve analysis as one peak at the expected melting temperature and by agarose gel electrophoresis.

### Intracranial AAV microinfusion procedure

Rats (*N* = 46) were anesthetized with 3–5% isoflurane and placed in a stereotaxic frame (Kopf Instruments) for bilateral AAV microinfusion into the CeA. Briefly, a 2 μl, 22-gauge Hamilton microsyringe was lowered 8.4 mm from skull surface into the CeA (AP −2.64, ML ±4.2) with the incisor bar set at −3.3 mm below the interaural line (flat skull), according to the Paxino’s and Watson’s rat brain atlas (Paxinos and Watson, 2007). Either a PAC1R knock-down adeno-associated viral vector (AAV1-CAG-GFP-rADCYAP1R1-shRNAmir, “AAV-PAC1R-shRNA”) or a Ctrl. virus (AAV1-CAG-GFP, “AAV-GFP”) was infused at a rate of 0.2 μl/min over the course of 5 min (total volume: 1 μl per side). The needle was kept in place for an additional 10 min following infusion to prevent backflow. The ability of this specific AAV-shRNA construct to knock-down PAC1R expression in rats was previously confirmed (Minnig et al., 2021, 2022). After surgeries, rats were allowed at least three weeks of recovery before the start of the CSDS procedure to allow for maximum transfection. At the end of testing, viral placement and spread were verified in a blind manner as assessed by GFP signal; only rats with correct viral location and satisfactory spread in both sides of the CeA were included in the data analysis (14 rats were excluded). Body weights were recorded before surgery, before the start of the CSDS protocol, and then every 1–2 d during the 10 d of CSDS.

### Light-dark test

The light–dark transfer test was performed as described previously (Bourin and Hascoët, 2003), 14–18 h after the seventh CSDS session. The test apparatus was a Plexiglas rectangular box (50 × 50 cm) divided into two unequal compartments by a black partition with a small opening at the base. The smaller compartment (1/3) was kept dark (∼0 lx), while the larger compartment (2/3) was illuminated (20 lx) by a 75W light bulb located above. Rats were placed in the center of the dark compartment facing toward the partition at the beginning of the test, after seven consecutive defeats. The sessions were recorded and the latency to enter the light compartment as well as the percent (%) of time spent in the light compartment during the 10-min test were scored by individuals blind to the treatments.

### Plasma CORT measurement

Plasma levels of CORT were determined as previously described (Cottone et al., 2009; Fekete et al., 2011; Dore et al., 2013; Iemolo et al., 2016). Blood was sampled from the rats’ tails 14–18 h after the ninth CSDS session and collected in tubes containing 0.5 m EDTA, pH 8.0 (Invitrogen, ThermoFisher Scientific). Plasma was obtained after blood centrifugation, and it was stored at −80°C until levels of CORT-like immunoreactivity were determined using a commercially available radioimmunoassay kit, according to the manufacturer’s instructions (MP Biomedicals). Intraassay and interassay coefficients of variation were <10%.

### CRF IHC

Rats from the PAC1R KD experiment were euthanized 24 h after the last (10th) CSDS session. After transcardial perfusion, coronal 30-μm sections were cut on a cryostat, collected, and stored in cryoprotectant at −20°C. Every sixth section (180 μm apart) of the CeA (bregma range: −2.0 to −3.0 mm) were processed for IHC. Slices were pretreated with 100 mm urea (pH 9.5) for 10 min at 95°C followed by 10 min in an iced water bath. Sections were placed for 1 h in blocking solution (3% normal donkey serum, 0.4% Triton X-100) and subsequently incubated overnight at room temperature with a cocktail of two primary antibodies in blocking solution, an anti-CRF (1:200, Santa Cruz) and an anti-GFP (1:1500, Abcam). Sections were then incubated with the secondary antibodies donkey anti-rabbit Alexa Fluor 488 and donkey anti-goat Cy3 (Jackson ImmunoResearch) 1:400 in blocking solution for 2 h at room temperature. Sections were mounted onto glass slides, coverslipped with Vectashield mounting medium (Vector Laboratories), and stored at 4°C.

#### Quantification of staining

Pictures of sections containing the CeL, where CRF immunoreactivity is mostly concentrated in, were taken as described above. CRF immunofluorescence pictures were captured through the Texas Red Filter cube (Olympus) under a preset exposure and gain. Densitometry analyses were performed using ImageJ software (NIH); images were converted to 8-bit and adjusted using the auto threshold Triangle algorithm. Once converted, mean density of the tracing for immunohistochemical signal was calculated and normalized based on the size of the tracing area.

### Statistical analysis

Three-way ANOVA was used on body weight change data and on light-dark test data, with Defeat and AAV-shRNA as between-subject factors and Time as a within-subject factor. Two-way ANOVAs were used on CRF density data, with Defeat and AAV-shRNA as between-subject factors. Pairwise *post hoc* comparisons were made using Newman–Keuls test; Student’s *t* test was used when comparing two groups. Significance was set at *p *≤* *0.05. The software/graphic packages used were SigmaPlot 11.0 and Statistica 7.0.

## Results

### Effects of CSDS on PACAP levels in the CeA and BNST

**Figure 1.**
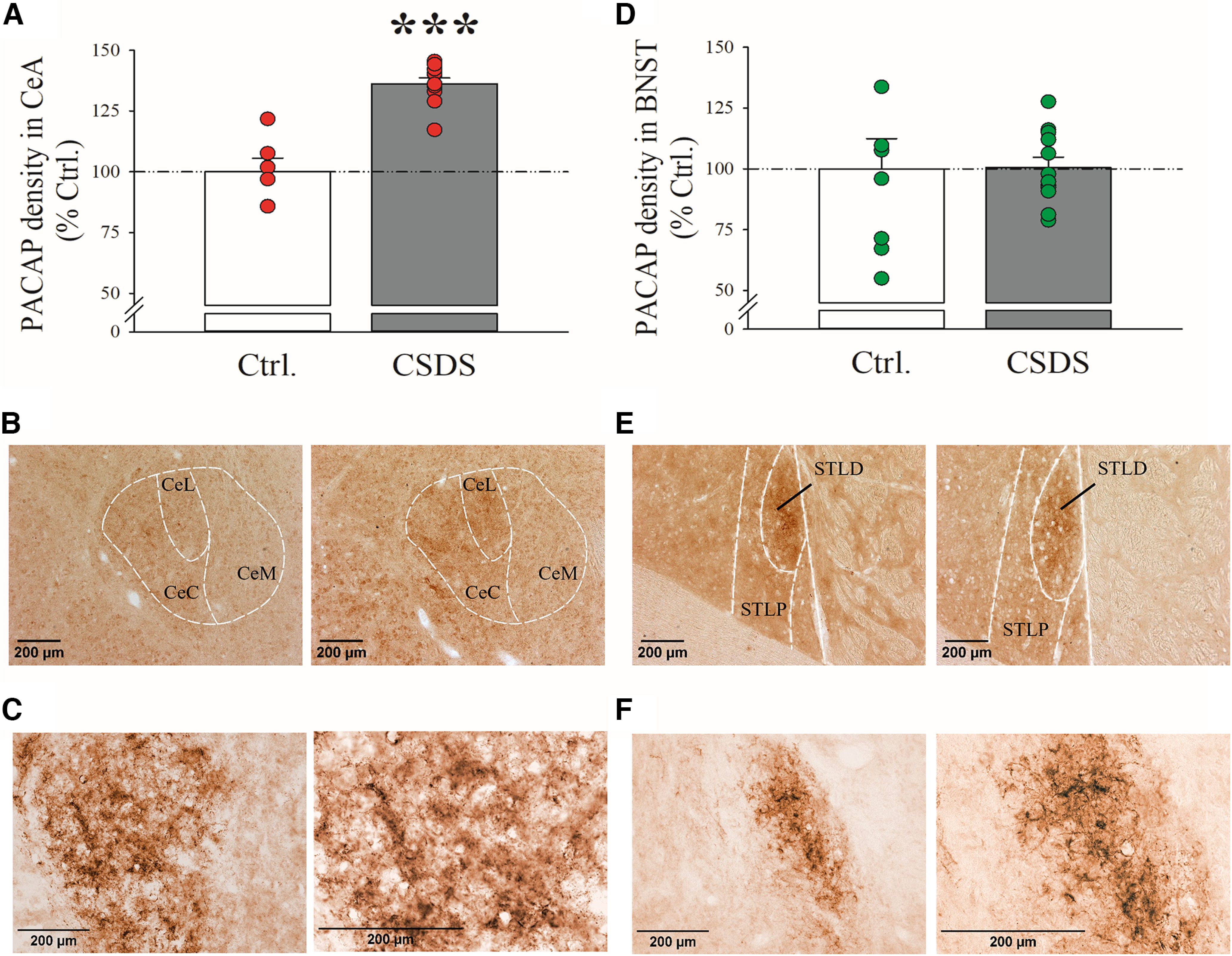
Rats were subject to 10 daily consecutive sessions of either a CSDS procedure or a Ctrl. procedure, and brains were collected 24 h after the last session for PACAP IHC in (***A*–*C***) CeA (CeC/CeL) and (***D*–*F***) BNST (STLD). *N* = 6–11/group. Representative 10× images of the staining in the (***B***) CeA and (***E***) BNST of Ctrl. and CSDS animals. 20× (left) and 40× (right) images of a representative PACAP staining in (***C***) CeA and (***F***) BNST. Bars represent mean ± SEM; ***p <* *0.001 versus Ctrl. CeA: CeC, capsular part; CeL, lateral part; CeM, medial part of the CeA. BNST: STLP, lateral division posterior part; STLD, lateral division dorsal part of the BNST. Extended Data Figure 1-1 shows the effects of 10 d of CSDS on PACAP immunoreactivity in the PVN. Extended Data Figure 1-2 shows the effects of a single SDS session on PACAP immunoreactivity in the CeA and BNST.

Rats were subject to 10 daily consecutive sessions of either a CSDS procedure or a Ctrl. procedure, and brains were collected 24 h after the last session for PACAP IHC. As shown in Figure 1*A*, CSDS caused a significant increase in PACAP levels (immunoreactivity) in the CeA (*t*_(15)_ = −6.93, *p* ≤ 0.001). Indeed, using densitometry, we found that CSDS animals showed a 23.3% increase in PACAP levels in the CeA, compared with nondefeated, Ctrl. animals. Conversely, CSDS did not alter PACAP levels in the BNST [*t*_(18)_ = 0.05, not significant (n.s.)], as shown in Figure 1*C* (0.6% increase). Interestingly, CSDS significantly reduced PACAP levels in the PVN (*t*_(18)_ = 3.62, *p* ≤ 0.01; Extended Data Fig. 1-1). Representative images of PACAP immunoreactivity are shown in Figure 1*B*,*C*,*E*,*F*. The PACAP immunoreactivity in the CeA consists of fibers and is restricted to CeC and CeL, while in the BNST fiber staining is restricted to BSTLD, and therefore these were the subdivisions quantified. Interestingly, a single SDS increased PACAP levels in both CeA and BNST (Extended Data Fig. 1-2). Briefly, when a cohort of rats (*N* = 8–9/group) was subject to a single social defeat session (single SDS) or a Ctrl. procedure, and PACAP immunoreactivity assessed 24 h later, a Single SDS was found to cause a significant increase in PACAP immunoreactivity in both the CeC/CeL (*t*_(15)_ = −3.98, *p* ≤ 0.001, 47.2%; Extended Data Fig. 1-2*A*) and the STLD (*t*_(14)_ = −2.72, *p* ≤ 0.05, 27.5%; Extended Data Fig. 1-2*B*).

10.1523/ENEURO.0260-22.2022.f1-1 Extended Data Figure 1-1 Effects of 10 days of CSDS on PACAP immunoreactivity in the (A) PVN. N= 8-12/group). (B) Representative 10x images of the staining in the PVN of Ctrl. and CSDS animals. Bars represent Mean ± SEM. ** p < 0.01 vs. Ctrl. PVN: paraventricular nucleus of the hypothalamus. Download Figure 1-1, TIF file.

**Figure 2.**
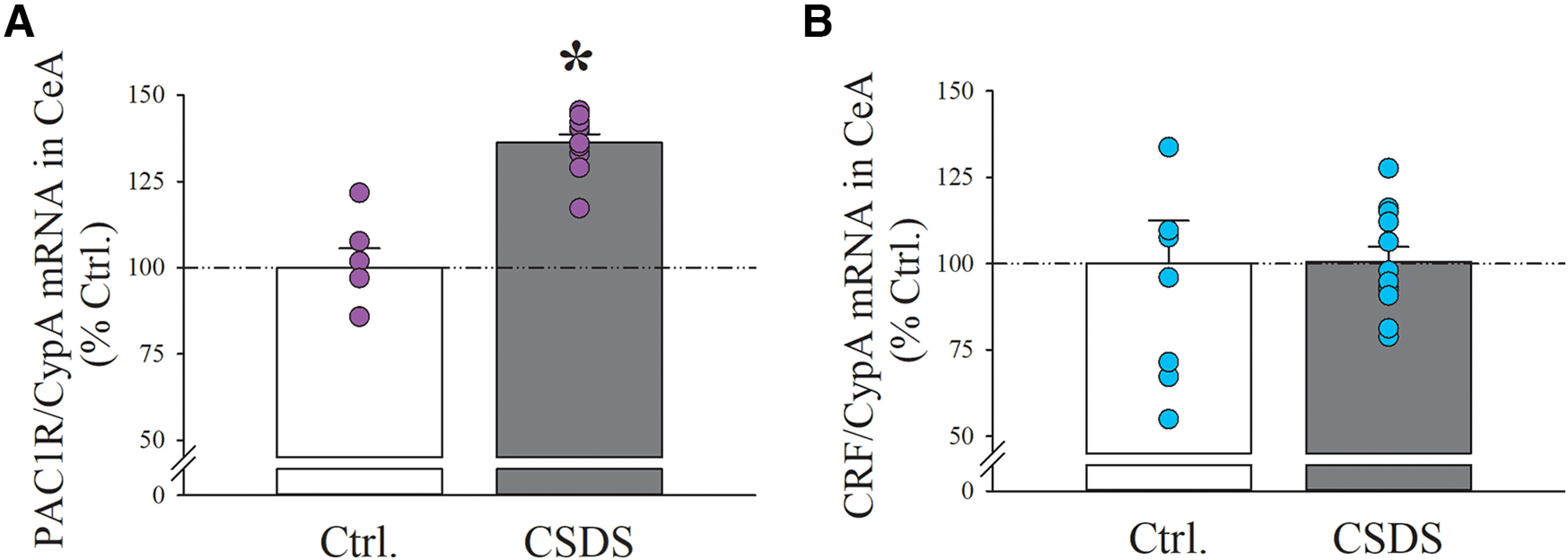
Rats were subject to 10 daily consecutive sessions of either a CSDS procedure or a Ctrl. procedure, and brain punches containing the CeA were collected 24 h after the last session for assessment of gene expression using qPCR: (***A***) PAC1R and (***B***) CRF mRNA levels in the CeA. *N* = 6–11/group for PAC1R, 8–12/group for CRF. Bars represent mean ± SEM; *p <* *0.05 versus Ctrl.

10.1523/ENEURO.0260-22.2022.f1-2 Extended Data Figure 1-2 Effects of 1 single social defeat session (Single SDS) on PACAP immunoreactivity in the CeA (A) and BNST (B) of rats. N= 8-9/group. Bars represent Mean ± SEM. ** p < 0.01, *** p < 0.001 vs. Ctrl. Download Figure 1-2, TIF file.

### Effects of CSDS on PAC1R and CRF mRNA levels in the CeA

A separate set of rats was subject to 10 daily consecutive sessions of either a CSDS procedure or a Ctrl. procedure, and brain punches containing the CeA were collected 24 h after the last CSDS session. Using qPCR, we found that CSDS rats display higher levels of PAC1R mRNA in CeA, compared with Ctrl. rats (+31.9%, *t*_(16)_ = 2.11, *p* < 0.05) CRF mRNA in CeA was found to be unaffected by CSDS (*t*_(16)_ = 0.06, n.s.). We did not quantify the PACAP transcript because PACAP is not synthetized in this region and fibers are of nonlocal origin.

### PAC1R knock-down in the CeA attenuates chronic social defeat-induced reduction in body weight gain

**Figure 3.**
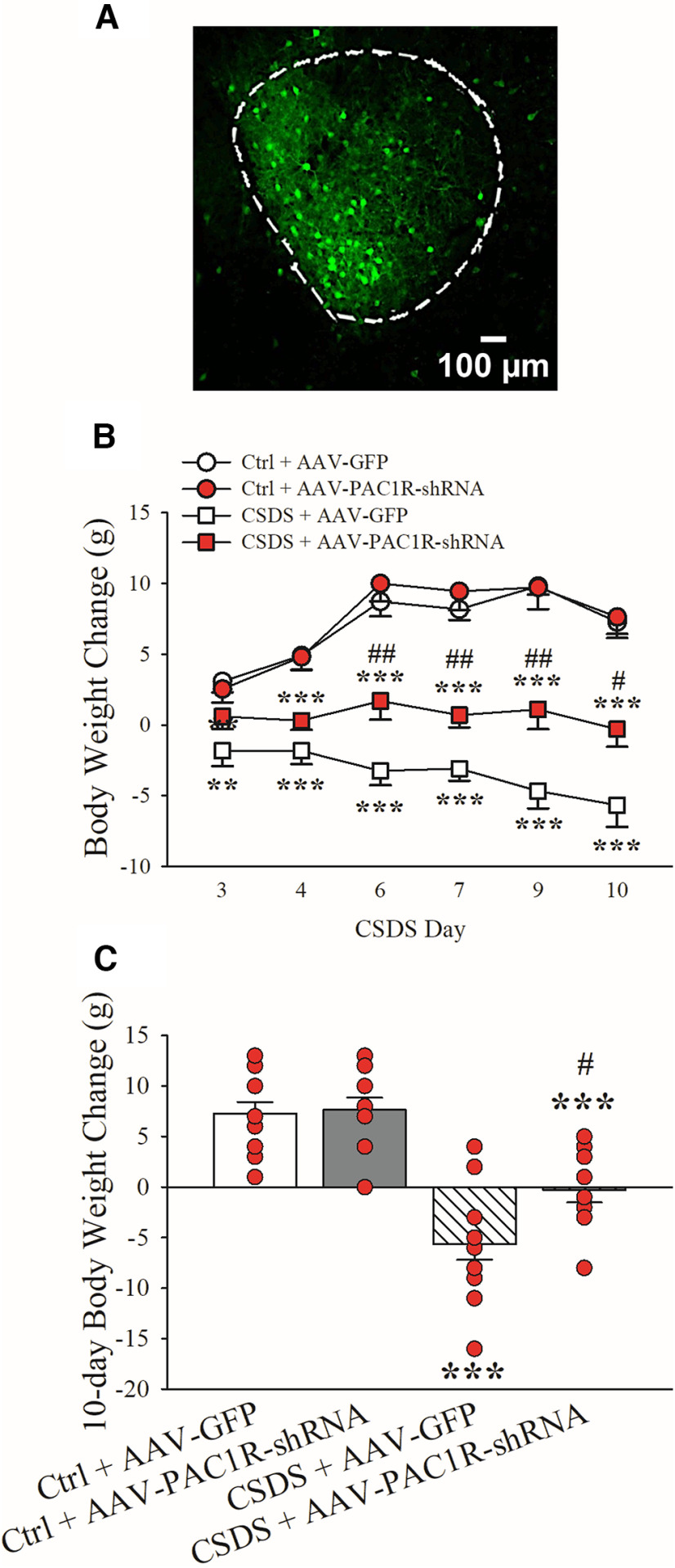
Rats were bilaterally microinfused into the CeA either a PAC1R knock-down adeno-associated viral vector (AAV-PAC1R-shRNA) or a Ctrl. virus (AAV-GFP), and body weight was recorded every 1–2 d over the course of the CSDS (or Ctrl.) paradigm. ***A***, Representative image of viral spread in CeA (GFP). Effect of bilateral CeA PAC1R knock-down on (***B***) body weight change across days and (***C***) cumulative 10-d body weight change. *N* = 9–12/group. Bars represent mean ± SEM; **p ≤* *0.01, ***p ≤* *0.001 versus Ctrl. + AAV-GFP; #*p *≤* *0.05, ##*p *≤* *0.01 versus CSDS + AAV-GFP.

Before the beginning of the CSDS paradigm (i.e., 26–33 d after AAV infusion), body weight did not significantly differ between the AAV-GFP and AAV-PAC1R-shRNA group (average ± SEM, AAV-GFP: 376.0 ± 3.4 g, AAV-PAC1R-shRNA: 378.8 ± 3.1 g; *t*_(44)_ = 0.61, n.s.). Figure 3*A* shows a representative viral spread in the CeA. As shown in Figure 3*B*, in animals infused with a Ctrl. AAV (GFP groups), CSDS induced a reduction in body weight gain (white squares), compared with nondefeated, Ctrl. animals (white circles; CSDS: *F*_(1,39)_ = 100.98, *p* ≤ 0.001). Knocking down PAC1R in the CeA three weeks before the start of the CSDS procedure was able to significantly attenuate the CSDS-induced reduction in body weight across the entire 10 d defeat period (red squares, CSDS + AAV-PAC1R-shRNA) compared with CSDS-GFP, without affecting body weight change in nondefeated, Ctrl. animals (red circles, Ctrl. + AAV-PAC1R; AAV Type × CSDS: *F*_(1,39)_ = 5.38, *p* ≤ 0.05; AAV Type: *F*_(1,39)_ = 7.57, *p* ≤ 0.01). Figure 3*C* shows the cumulative body weight gain of the four groups of animals in the 10-d period.

### Effects of PAC1R knock-down in the CeA on CSDS-induced anxiety-like behavior

**Figure 4.**
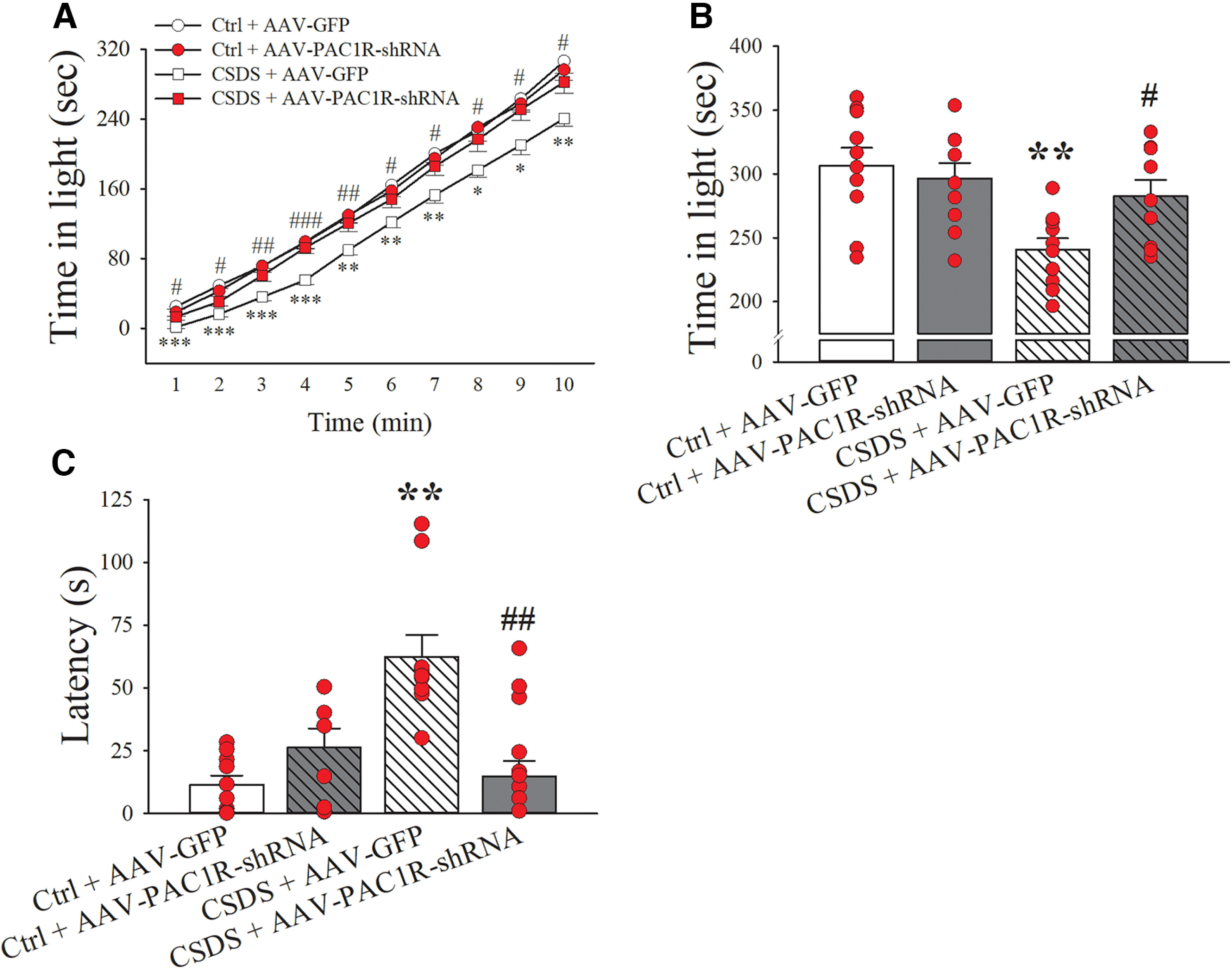
AAV-GFP and AAV-PAC1R-shRNA rats were subject to either CSDS or a Ctrl. procedure to assess the effects of bilateral CeA PAC1R knock-down on anxiety-like behavior in the light-dark box test on (***A***) time spent in the light compartment across time, (***B***) total time spent in the light compartment in the 10-min test, and (***C***) latency to first leave the dark compartment. *N* = 9–10/group. Bars represent mean ± SEM; *p ≤* *0.05, **p ≤* *0.01, ***p ≤* *0.001 versus Ctrl. + AAV-GFP; #*p *≤* *0.05, ##*p *≤* *0.01, ###*p *≤* *0.001 versus CSDS + AAV-GFP.

CSDS induced anxiety-like behavior, as measured by a reduction in time spent in the light compartment of a light-dark test box in CSDS-GFP animals compared with Ctrl-GFP animals, as shown in the time course in Figure 4*A*. Knock-down of PAC1R in the CeA was able to reverse this heightened anxiety across the 10 min of the test (AAV Type × CSDS: *F*_(1,35)_ = 4.65, *p* ≤ 0.05). Indeed, CSDS + AAV-PAC1R-shRNA animals spent significantly more time in the light compartment compared with CSDS + AAV-GFP animals and were no different from the Ctrl. + AAV-GFP group. Figure 4*B* shows the time spent in the light compartment by the four groups of animals in the cumulative 10 min.

Furthermore, knock-down of PAC1R in the CeA significantly decreased the latency to first exit the dark compartment of the box induced by chronic social defeat, as shown in Figure 4*C* (AAV Type × CSDS: *F*_(1,35)_ = 8.65, *p* ≤ 0.01).

### Effects of PAC1R knock-down in the CeA on plasma CORT levels

**Figure 5.**
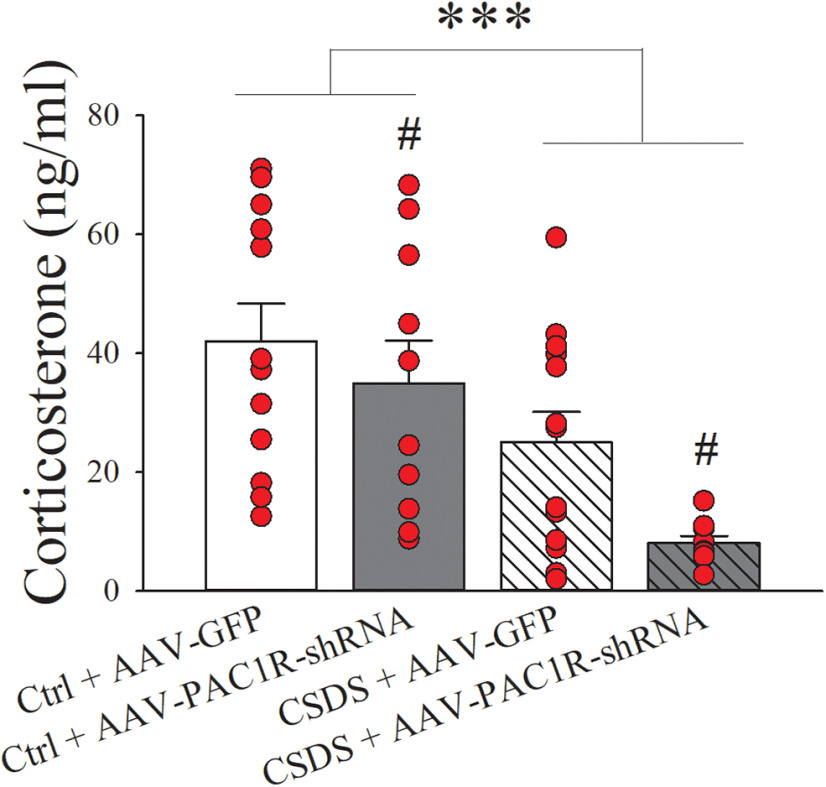
AAV-GFP and AAV-PAC1R-shRNA rats were subject to either CSDS or a Ctrl. procedure to assess the effects of bilateral CeA PAC1R knock-down on plasma CORT levels. *N* = 9–12/group. Bars represent mean ± SEM; ***p < 0.001 versus Ctrl groups; #*p *≤* *0.05 versus AAV-GFP groups.

CSDS induced a pronounced reduction in plasma circulating CORT levels (CSDS: *F*_(1,40)_ = 14.74, *p* ≤ 0.001) and knock-down of PAC1R in the CeA caused a further reduction in CORT levels (AAV Type: *F*_(1,40)_ = 4.43, *p* ≤ 0.05), as shown in Figure 5. PAC1R knock-down did not differentially affect CORT levels in the Ctrl. and CSDS group regardless of CSDS exposure (AAV type × CSDS: *F*_(1,40)_ = 0.75, n.s.). Indeed, the plasma CORT concentration for the Ctrl. + AAV-GFP group was 42.1 ± 6.3 ng/ml, this value was 25.1 ± 5.1 ng/ml in the CSDS + AAV-GFP group and the lowest (8.1 ± 1.2 ng/ml) in the CSDS + AAV-PAC1R-shRNA group.

### Effects of PAC1R knock-down in the CeA on CSDS-induced increases in CRF

**Figure 6.**
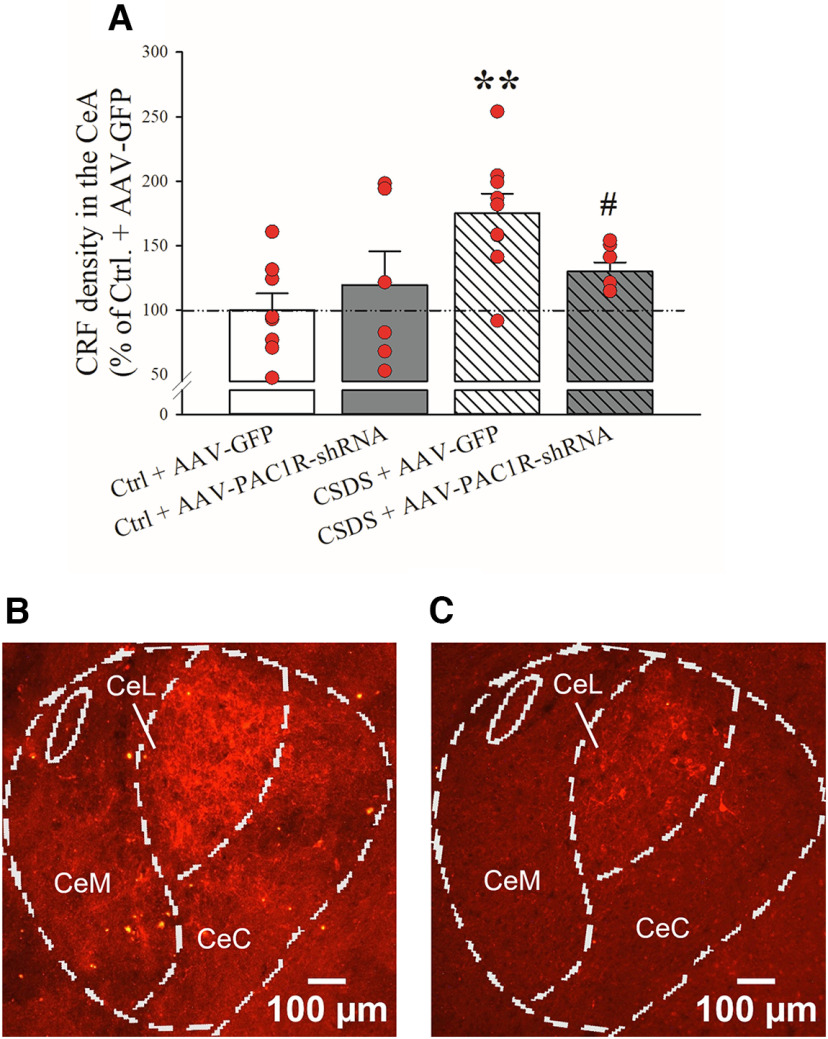
AAV-GFP and AAV-PAC1R-shRNA rats were subject to either CSDS or a Ctrl. procedure and were then euthanized to assess the effects of bilateral CeA PAC1R knock-down on (***A***) CRF immunoreactivity in the CeL (optical density). *N* = 6–9/group. Representative CRF staining in the CSDS + AAV-GFP and CSDS + AAV-PAC1R groups (***B***, ***C***). Bars represent mean ± SEM; **p ≤* *0.01 versus Ctrl. +AAV-GFP; #*p *≤* *0.05 versus CSDS + AAV-GFP. CeC, capsular part; CeL, lateral part; CeM, medial part of the CeA.

Rats infused with either AAV-GFP or AAV-PAC1R-shRNA and subject to either CSDS or a Ctrl. procedure were euthanized 24 h after the last CSDS session and the brains collected for CRF IHC. As shown in Figure 6*A*, CSDS caused an increase in CRF immunoreactivity in the CeA (Defeat: *F*_(1,26)_ = 7.30, *p* ≤ 0.05). However, the knock-down of PAC1R in the CeA significantly attenuated social defeat-induced increase in CRF (AAV-PAC1R × CSDS: *F*_(1,26)_ = 4.12, *p* ≤ 0.05). Representative images of CRF IHC in the CeA are shown for the CSDS-GFP (Fig. 6*B*) and the CSDS-AAV-PAC1R group (Fig. 6*C*).

## Discussion

Our findings were the following: (1) PACAP levels were increased in the CeA, but not the BNST, following CSDS; (2) reducing PAC1R levels in the CeA via a viral vector containing a short hairpin RNA significantly attenuated CSDS-included body weight loss; (3) CeA PAC1R knock-down abolished CSDS-induced heightened anxiety-like behavior; (4) CeA PAC1R knock-down prevented CSDS-induced local increase in CRF levels. Collectively, the results of the present study reveal an important role for PACAP and PAC1R of the CeA in regulating the physiological and behavioral responses to chronic psychosocial stress.

The finding that PACAP immunoreactivity levels is higher in the CeA, but not in the BNST, of CSDS rats is a significant one as, to our knowledge, selective increases in CeA PACAP levels as a result of any type of chronic stress have not been reported before. This increase was observed 24 h after the last social defeat session, suggesting that the effect does not dissipate shortly after the end of the defeat session. Notably, a single SDS session caused an elevation in PACAP levels in both CeA and BNST (see Extended Data Fig. 1-2), in line with previous reports showing that a single 10-min footshock session increases PACAP immunoreactivity in both brain regions (Seiglie et al., 2019). While the PACAP increase in BNST following acute SDS appears, therefore, to be transitory in nature and to undergo habituation with repeated sessions, the PACAP increase in CeA is instead persistent, suggesting that CeA PACAP recruitment could mediate the effects of CSDS. In this study, PACAP immunoreactivity in both the CeA and BNST appeared as fibers and cell bodies positive for the peptide were not visible, as reported before (Köves et al., 1991; Piggins et al., 1996; Hannibal, 2002; Seiglie et al., 2019). In line with the lack of PACAP mRNA in these regions, the majority of these fibers have been proposed to represent afferent projections from other brain areas, and in particular the lateral parabrachial nucleus (lPBn), a critical source of the peptide in CeA and BNST (Missig et al., 2014, 2017). Interestingly, the chemogenetic activation lPBn-BNST PACAP projection has recently been shown to enhance anxiety-like behavior (Boucher et al., 2021b), while the effects of the direct stimulation of the lPBn-CeA PACAP pathway has not yet been reported. Importantly, we found using qRT-PCR that CSDS significantly increased PAC1R levels in the CeA, suggesting that chronic exposure to this psychosocial stress also upregulate the receptor.

To test the functional relevance of CSDS-induced increase PACAP levels and increase in PAC1R expression in CeA, a viral vector approach was used to test the effects of knocking down PACAP selective receptor PAC1R, which is highly expressed in this area (Joo et al., 2004), on the outcomes of CSDS. Stress can have profound effects on body weight. Acute and chronic stressors inhibit food intake and cause significant weight loss (Krahn et al., 1990; Martí et al., 1994). In this study, CSDS reduced body weight gain throughout the stress exposure period, consistent with previous findings (Bhatnagar et al., 2006; Krishnan et al., 2007; Becker et al., 2008; Pulliam et al., 2010; Venzala et al., 2012). We found that CeA PAC1R knock-down significantly attenuated the stress-induced reduction in body weight gain, suggesting that the PACAP released in CeA following CSDS contributes to the reduced body weight gain. Notably, the PAC1R knock-down had no effect on body weight gain in Ctrl., unstressed rats, indicating that the role of the PAC1R system in this specific brain region in the regulation of body weight is specific to changes because of the stressors, rather than pure ingestive behavior/metabolism. Although we did not measure food intake in this study, previous studies have shown that, in models of chronic stress, the reduction in body weight gain is a result of stress-induced hypophagia (Iio et al., 2012, 2014). Our data showing a role of PACAP in stress-induced body weight changes are in line with a previous study showing that chronic administration of the PAC1R/VPAC2R antagonist PACAP(6–38) is able to block chronic variable stress-induced changes in weight gain (Roman et al., 2014), as well as with another study showing that PACAP knock-out mice are significantly protected from the effects of chronic restraint stress on weight loss (Mustafa et al., 2015). The induction of anorexia is a well-documented effect of PACAP (Mounien et al., 2009; Dore et al., 2013; Resch et al., 2013; Kocho-Schellenberg et al., 2014); our results are consistent in particular with previous report that intra-CeA infusion of PACAP causes anorexia and reduced body weight gain (Iemolo et al., 2015).

The mechanism by which PAC1R blockade counteracts body weight loss is not completely clear. Exogenous CRF administration suppresses food intake and its release during stress contributes to stress-induced hypophagia via activation of CRFR1, as shown by the ability of CRFR1 antagonists to block stress-induced inhibition of feeding behavior (Smagin et al., 1999; Griebel et al., 2002; Chotiwat and Harris, 2008). Based on these actions of CRF, and considering that PACAP has been shown to be upstream of CRF for several of its actions (Tachibana et al., 2003; Maruyama et al., 2006; Dore et al., 2013), we can speculate that the effect of PAC1R knock-down on CSDS-induced body weight change may involve the inhibition of the CRF/CRFR1 system. However, it is worth noting that the anorexigenic effect of PACAP in nonstressed conditions does not appear to involve CRF (Dore et al., 2013; Iemolo et al., 2015). Another possible mechanism could be the inhibition of the melanocortin and BDNF systems, as PACAP’s anorectic effects have been shown to involve the activation of MC4R and TrkB in the CeA (Iemolo et al., 2015). PAC1R knock-down in the CeA did not, however, completely block the effects of CSDS on body weight change, suggesting that other brain areas and/or other systems are likely also involved in this phenomenon, or that the degree of PAC1R knock-down attained in CeA was insufficient to observe a full reversal.

CSDS has large effects on behavior. In general, defeated animals show signs of lower wellbeing, including a heightened anxiety-like state, as measured with a variety of tests (Kinsey et al., 2007; Bailey et al., 2009; Wohleb et al., 2011; Hanke et al., 2012; Iñiguez et al., 2014; Macedo et al., 2018). Here, we observed that CSDS induced anxiety-like behavior, as evidenced by reduced time spent in the light compartment of a light-dark box and of the increased latency to first exit the dark compartment. The light-dark test is based on an approach-avoidance conflict between exploration of a novel environment and avoidance of brightly lit, open spaces and it is sensitive to states of stress as well as anxiogenic/anxiolytic drugs (Crawley and Goodwin, 1980; Crawley, 1985; Merlo Pich and Samanin, 1989; Young and Johnson, 1991; Chaouloff et al., 1997). PAC1R knock-down in the CeA was able to prevent this CSDS-induced anxiety-like behavior, suggesting that CeA PAC1R activation mediates this behavioral effect of CSDS. Our results are in line with previous observations with whole body PACAP deletion, showing that CSDS-exposed PACAP knock-out mice have markedly attenuated CSDS-induced emotional deficits, compared with wild-type Ctrl. mice (Lehmann et al., 2013). PAC1R knock-down in the CeA had no effect on anxiety-like behavior in Ctrl., unstressed rats. This result is consistent with the profile observed with PAC1R antagonists in previous studies (Seiglie et al., 2019) and suggest that endogenous PACAP is not released in CeA under basal, unstressed condition, and that instead this system becomes activated in response to a high-intensity or chronic stress. This profile shows similarities with CRFR1 antagonists, which display efficacy in exploration-based models of anxiety under stressed, but not in nonstressed testing conditions (Okuyama et al., 1999; Gilligan et al., 2000; Griebel et al., 2002; Heinrichs et al., 2002; Zorrilla et al., 2002; Lelas et al., 2004; Ising et al., 2007; Zorrilla and Koob, 2010). The viral vector approach has clear advantages over the classical pharmacological approach in this specific case, in that it allows to skip the issue of poor selectivity of available PAC1R antagonists and allows to reach a constant blockade of PAC1R during the course of the CSDS exposure, without the need for repeated intracranial injections.

We also assessed the effect of CeA PAC1R knock-down on plasma CORT levels in both unstressed and CSDS animals. We found lower basal CORT levels in CSDS rats compared with Ctrls., is in line with previous preclinical reports showing reduced baseline CORT levels and blunted hypothalamic pituitary adrenal (HPA) axis reactivity following CSDS, predator exposure models, and immobilization paradigms (Liberzon et al., 1997; Beitia et al., 2005; Harvey et al., 2006; Arndt et al., 2009; Zoladz et al., 2015), and consistent with what observed clinically in PTSD patients (Yehuda et al., 1993; Yehuda, 2001). The hyporeactive HPA axis characteristic of PTSD is thought to be because of enhanced negative feedback sensitivity via increased glucocorticoid receptor responsiveness (Yehuda et al., 2009; Hartmann et al., 2012; Schöner et al., 2017). Notably, the reduced basal CORT levels in CSDS rats may be in agreement with the reduced PACAP levels we found in the PVN; since PACAP in this brain region has been shown to mediate stress-induced activation of the HPA axis as well as elevations in CRF mRNA (Stroth and Eiden, 2010; Lehmann et al., 2013), PACAP reductions in PVN by CSDS may be responsible for the lower basal CORT levels. Interestingly, CeA PAC1R knock-down further decreased basal CORT levels, in both unstressed and CSDS rats. While this is in line with previous report that exogenous intra-CeA administration of PACAP elevates plasma CORT levels (Iemolo et al., 2016); it also suggests that the reversal of the CSDS-induced heightened anxiety-like behavior is not a consequence of its effects on the HPA axis, as PAC1R knock-down did not “normalize” CORT levels in CSDS animals, but rather a cumulative effect of CSDS and CeA PAC1R knock-down was observed. These results are in agreement with the notion that the behavioral response to stress is mediated by the extended amygdala and occurs independently of HPA axis activation (Britton et al., 1986; Dunn and Berridge, 1990; Menzaghi et al., 1994; Koob and Heinrichs, 1999) and with previous findings that the anxiogenic and the HPA activating effects of PACAP administration involve different mechanisms (Dore et al., 2013).

The CeA is a very heterogeneous structure with a rich diversity of cell types and complex circuitry (Ciocchi et al., 2010; Haubensak et al., 2010; Ahrens et al., 2018; McCullough et al., 2018), and the PACAP neurocircuit mechanisms in the CeA are so far not well understood. Using IHC, we found that CSDS resulted in a significant increase in CRF levels in the CeA. This observation is in line with previous findings showing elevated CRF and elevated CRF receptor binding in the CeA following chronic psychosocial stress (Fuchs and Flügge, 1995; Albeck et al., 1997). PAC1R knock-down in the CeA was able to significantly prevent the increases in CRF levels caused by CSDS, suggesting that CRF activation may be the downstream mechanism mediating the detrimental effect of PAC1R upregulation and hyperactivity. While CSDS-GFP animals had about a 75% increase in CeA CRF compared with their nonstressed counterparts, CSDS-PAC1R knock-down rats had only a 10% increase from their nonstressed counterparts and, most striking, 45% less CRF in the CeA compared with the defeated-GFP rats. CeA PAC1R knock-down had no effect on CeA CRF levels in nonstressed Ctrls. CRF in the CeA is expressed both in neuronal cell bodies made locally as well as in terminals originating also from afferent brain regions; since our data showed that CSDS does not affect CeA CRF mRNA, we focused on CRF terminals and used densitometry to quantify CRF staining, as fiber staining is very evident and limited CRF-immunoreactive neurons can be detected unless animals are previously treated with an axonal transport blocker (Wang et al., 2011). Central administration of PACAP has been shown to augment CRF expression and CRF neuronal activation in the hypothalamus (Grinevich et al., 1997; Li and Sawchenko, 1998; Agarwal et al., 2005; Norrholm et al., 2005) as well as CRF peptide levels in the CeA (Dore et al., 2013), whole-body PACAP deletion prevents the increase of CRF expression by prolonged stress (Stroth and Eiden, 2010). We speculate that PACAP may affect local CRF release via a presynaptic action on CRF terminals in the CeA. In support of the former hypothesis, Varodayan et al. (2020) found that, in the medial subdivision of CeA (CeM), PACAP increases CeM GABA signaling via a presynaptic mechanism of action on PAC1R which, in microcircuits containing multiple GABAergic neurons, can result in a disinhibition of inhibitory neurons and an increase in net CeM stimulatory output. Interestingly, a similar presynaptic increase in GABA release has been shown for CRF in the same model (Cottone et al., 2009; Roberto et al., 2010; Cruz et al., 2012; Varodayan et al., 2017). Therefore, our results are in line with a proposed involvement of the local CRF system in the effects of PACAP in the CeA, although future experiments will need to test this hypothesis directly. Another hypothesis that could be tested in future studies is that PACAP released in CeA during stress may activate the PKCδ neuronal population, whose activation elicits aversion, anxiety, and nociception (Cai et al., 2014; Botta et al., 2015; Wilson et al., 2019; Chen et al., 2022). Future experiments will be needed to determine the cell-types and circuits activated by PACAP, to better understand how this neuropeptide fits into this structural and functional complexity.

These results point to a key role of CeA, and not BNST, in the effect of this specific type of chronic stressor. Although the areas activated following social defeat have been described, the role of specific brain areas in CSDS-induced emotional dysregulation remains unclear. Specifically in the context of the extended amygdala, it has been proposed that chronic stress, and the anxiety-related behaviors resulting from it, is related more to the functioning of the BNST than the amygdala (Davis et al., 1997; Walker et al., 2009; Ressler, 2010). Our data suggest instead that this notion that CeA is involved in short-term, phasic fear while the BNST would mediate sustained fear may not always be accurate, perhaps depending on the specific type and pattern of stress (and, accordingly, while here CSDS selectively increases PACAP in CeA, chronic variable stress has been shown to selectively increase PACAP in BNST; Hammack et al., 2009; Lezak et al., 2014). Our results are consistent with the extensive preclinical and human literature suggesting that a hyperreactive amygdala is key to an exaggerated perception of the threat, and to anxiety and mood disorders in general (Roozendaal et al., 1997; Whalen et al., 2001; Davidson et al., 2002; Frodl et al., 2008; Admon et al., 2009; Pitman et al., 2012). In addition, circuits mediating anxiety/aversion versus fear conditioning are recently beginning to be differentiated, which may have played a role in the apparent discrepancy (Ciocchi et al., 2010; Haubensak et al., 2010; Cai et al., 2014; Meloni et al., 2019; Wilson et al., 2019; Chen et al., 2022). In addition, our data are a further demonstration that while the magnitude of the HPA stress response is limited by negative feedback mechanisms, the enhanced amygdala activity following chronic stress can trigger a positive feedback loop which potentiates anxiety and avoidance, therefore potentially promoting the development of stress-related pathologies (McEwen, 2017). A limitation of this study is that it was performed exclusively in male subjects because historically the CSDS model was developed in males; future studies will be needed to determine either the generalizability or the selectivity of the effects using female animals.

Altogether, these results suggest that chronic psychosocial stress recruits the PACAP/PAC1R system of the CeA in rats and that it mediates its negative physiological and behavioral consequences, independently of the HPA axis. Perturbations of the CeA PACAP-PAC1R system may, therefore, mediate the aberrant stress responses characteristic of anxiety-related disorders and PTSD, perhaps via a modulation of CRF release. PACAP and PAC1R represent potential important therapeutic targets for these psychopathologies.

## Synthesis

Reviewing Editor: Leandro Vendruscolo, National Institute on Drug Abuse Decisions are customarily a result of the Reviewing Editor and the peer reviewers coming together and discussing their recommendations until a consensus is reached. When revisions are invited, a fact-based synthesis statement explaining their decision and outlining what is needed to prepare a revision will be listed below. The following reviewer(s) agreed to reveal their identity: Florence Varodayan, Renata Marchette.

Reviewer 1

The pituitary adenylate cyclase-activating polypeptide (PACAP) system of the central amygdala mediates the detrimental effects of chronic social defeat stress in rats.

In this manuscript, the authors examined the role of central amygdala PACAP signaling in chronic social defeat stress in male rats. Using several complementary techniques (PCR, IHC, behavior, site specific viral strategy) they have identified several threads of evidence that support a critical role for PACAP/PAC1 signaling in mediating the physiological (body-weight loss) and behavioral (anxiety-like behavior) aspects of CSDS. Overall, this an interesting and well-written study. However, there are some concerns described below:

-In the discussion the authors claim that "The finding that PACAP immunoreactivity levels is higher in the CeA, but not in the BNST, of CSDS rats is a significant one as, to our knowledge, selective increases in CeA PACAP levels as a result of any type of chronic stress have not been reported before. This increase was observed 24 hr after the last social defeat session, suggesting that the effect does not dissipate shortly after the end of the defeat session... suggesting that CeA PACAP recruitment could mediate the effects of CSDS." To better support this claim, the authors should examine PACAP expression in the CeA 1-2 hr after the final CSDS session to ensure that there is no reduction/no change followed by a subsequent increase. See PMID: 31476352.

-The authors should show representative figures and quantify the amount of PAC1 knock-down they achieved in both nonstressed and CSDS groups using their viral approach.

Minor Concerns:

-There are some minor typos throughout the manuscript.

-It's not clear what company the rats were sourced from.

-The authors state that: "At the end of testing, viral placement and spread were verified in a blind manner as assessed by GFP signal, and only rats with correct viral location and satisfactory spread in both sides of the CeA were included in the data analysis." Please report the number of animals excluded due to these concerns.

-The authors should review their reported animal numbers. For example in Extended Data Figure 1.1, N=6-11 is reported, but both bars show more than 6 subjects. Also, please add a scale bar to the representative images in this figure.

-For Figure 3B, please indicate which group is represented by which symbol on the graph or in the figure legend.

Reviewer 2

The manuscript is well written and the results are interesting and advance the field. There are some points the authors need to address:

1- The use of only male rats needs to be addressed as a caveat of their study. Miczek, Gordon, and Russo groups have published social defeat protocols using female subjects.

2- In the methods the authors state that the animals were in direct contact with aggressors until submission, did the latency to submission change over time? What is the percentage of animals that had to be presented to more than one aggressor in a single session? Do these differences during the defeat sessions influence the behavioral and biochemical measures?

3- Why only data from days 3, 4, 6, 7, 9, and 10 are shown for body weight gain/loss?

4- Figure 4A seems superfluous.

5- The corticosterone data distribution in CSDS + GFP group appears to be bimodal, how does this relate to behavioral or other molecular and biochemical measures?

6- The figure legends should be more informative with detail on the timeline of each experiment and a brief procedure description.

## Author Response

Manuscript eN-NWR-0260-22

Detailed responses to Reviewers’ comments

We are very grateful to the Reviewers for all their comments to the manuscript and for their helpful, constructive criticisms. We are pleased that the Reviewers found our manuscript to be "interesting", “well-written", that “the results are interesting and advance the field”. We believe that we have addressed all the concerns raised and we are confident that the manuscript has been greatly improved thanks to these suggestions.

Reviewer #1

- “The authors should examine PACAP expression in the CeA 1-2 hr after the final CSDS session to ensure that there is no reduction/no change followed by a subsequent increase. See PMID: 31476352.”

We agree with Reviewer 1 that performing a full time-course of PACAP immunoreactivity levels after the last defeat would be potentially very interesting. However, we chose to assess PACAP levels 24 hr after the CSDS session, rather than acutely after, to be as close as possible to the time point of the behavioral measure (which occurred 14-18 hours after). Together with the finding that knockdown of PAC1R reverses the alterations in behavior, we, therefore, believe that the PACAP increase is the functionally relevant change for the effects of CSDS, although we certainly cannot exclude that fluctuations of PACAP levels may occur in acute vs. later time points. In addition, in PMID: 31476352 we showed that the immunoreactivity levels of PAC1R, rather than PACAP itself, were decreased acutely after a single restraint stress.

-The authors should show representative figures and quantify the amount of PAC1 knock-down they achieved in both nonstressed and CSDS groups using their viral approach.

We thank Reviewer 1 for the suggestion. We are afraid that we did not quantify PAC1R levels in the four conditions of Group 3 at the time this experiment was performed. We did assess the GFP location and spread in all animals, and we also collected some representative images of PAC1R and GFP staining, but we unfortunately did not systematically quantify them all.

-There are some minor typos throughout the manuscript.

The manuscript has been proof edited for typos.

-It’s not clear what company the rats were sourced from.

We apologize for the omission; the company has now been mentioned.

-The authors state that: “At the end of testing, viral placement and spread were verified in a blind manner as assessed by GFP signal, and only rats with correct viral location and satisfactory spread in both sides of the CeA were included in the data analysis.” Please report the number of animals excluded due to these concerns.

The number of animals excluded for unsatisfactory viral placement (14) has now been reported.

-The authors should review their reported animal numbers. For example in Extended Data Figure 1.1, N=6-11 is reported, but both bars show more than 6 subjects. Also, please add a scale bar to the representative images in this figure.

We apologize for the typo; the correct group size is now reported for Fig. 1-1 (N=8-12.group). We have now also added scale bars to the images.

-For Figure 3B, please indicate which group is represented by which symbol on the graph or in the figure legend.

We apologize for the omission; the complete legend is now shown in panel 3B.

Reviewer #2

1- The use of only male rats needs to be addressed as a caveat of their study. Miczek, Gordon, and Russo groups have published social defeat protocols using female subjects.

This limitation is now mentioned in the Discussion section.

2- In the methods the authors state that the animals were in direct contact with aggressors until submission, did the latency to submission change over time? What is the percentage of animals that had to be presented to more than one aggressor in a single session? Do these differences during the defeat sessions influence the behavioral and biochemical measures?

The residents were trained to become highly aggressive before the beginning of the CSDS study and, therefore, the latency was on average very low (60 sec or less) since the first session and did not change throughout the course of the CSDS. The number of animals that had to be presented to more than one aggressor in a single session was always <10% on a specific day, 0% most days.

3- Why only data from days 3, 4, 6, 7, 9, and 10 are shown for body weight gain/loss?

Unfortunately, body weight was not recorded daily in this experiment. This is specified in the Methods section.

4- Figure 4A seems superfluous.

We agree with Reviewer 2 that panels 4A and 4B are a bit redundant, but we respectfully think that it may be beneficial for the reader to appreciate the time-course of the difference in the anxiety test.

5- The corticosterone data distribution in CSDS + GFP group appears to be bimodal, how does this relate to behavioral or other molecular and biochemical measures?

We thank Reviewer 2 for the useful suggestion. We have now analyzed the data more in detail and found that the corticosterone data do not appear to correlate with any of the behavioral or molecular measures, in line with the observation that the effect of the shRNA on corticosterone level was not consistent with that on the rest of the measures (potentiation of the CSDS effect rather than reversal, and main effect of shRNA regardless of CSDS exposure). No statistically significant correlations were found in the AAV-GFP groups either, or among the other measures.

6- The figure legends should be more informative with detail on the timeline of each experiment and a brief procedure description.

All figure legends were updated to include more methodological information.
