# Supplementary figures and images for: The Pituitary Adenylate Cyclase-Activating Polypeptide (PACAP) System of the Central Amygdala Mediates the Detrimental Effects of Chronic Social Defeat Stress in Rats
Source: eNeuro. 2022 Sep 15;9(5):ENEURO.0260-22.2022. doi: 10.1523/ENEURO.0260-22.2022 (PMC9506682; doi:10.1523/ENEURO.0260-22.2022)

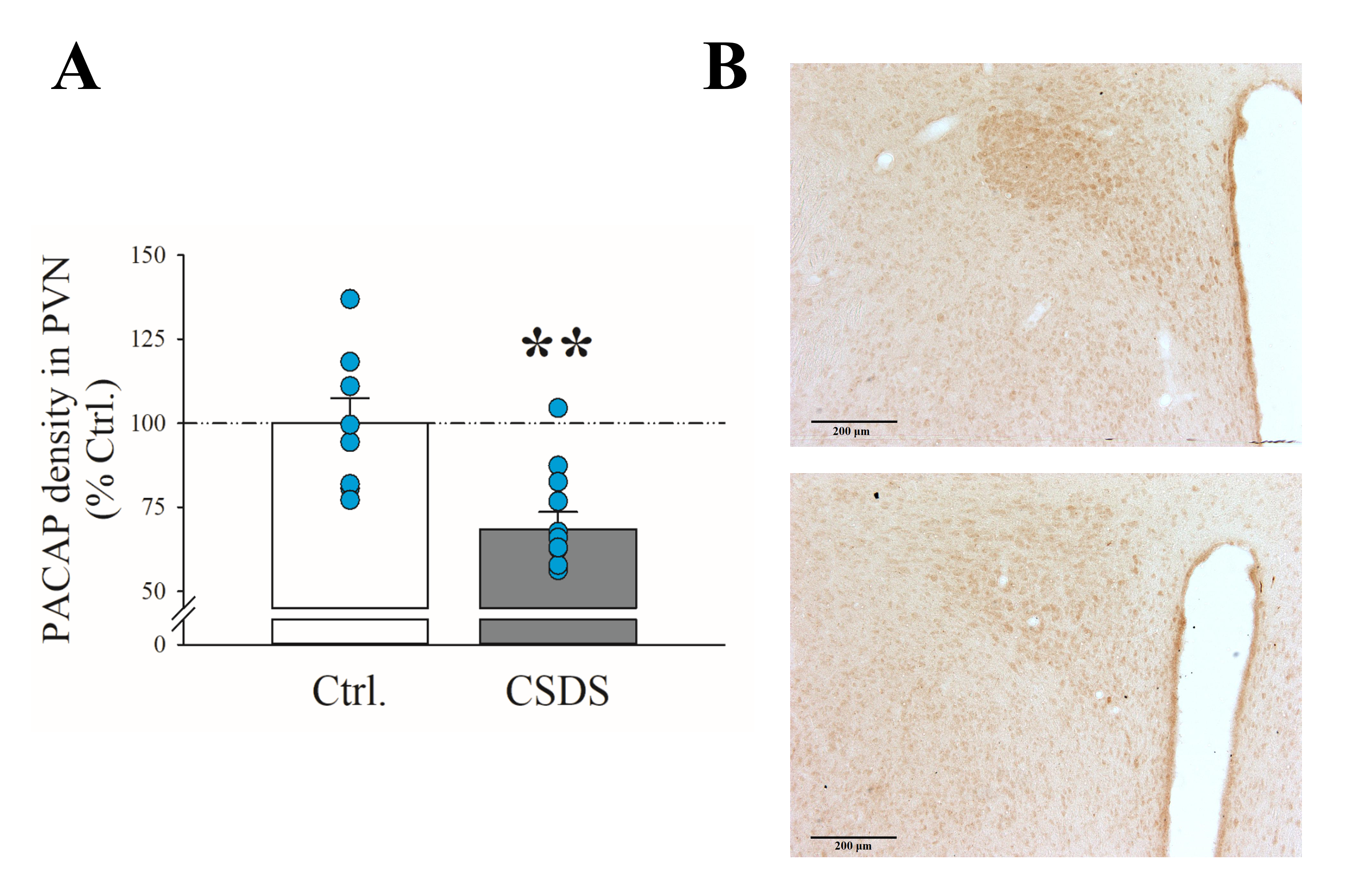

Supplement: Extended Data Figure 1-1 — Effects of 10 days of CSDS on PACAP immunoreactivity in the (A) PVN. N= 8-12/group). (B) Representative 10x images of the staining in the PVN of Ctrl. and CSDS animals. Bars represent Mean ± SEM. ** p < 0.01 vs. Ctrl. PVN: paraventricular nucleus of the hypothalamus. Download Figure 1-1, TIF file. [file enu-eN-NWR-0260-22-s02.tif]

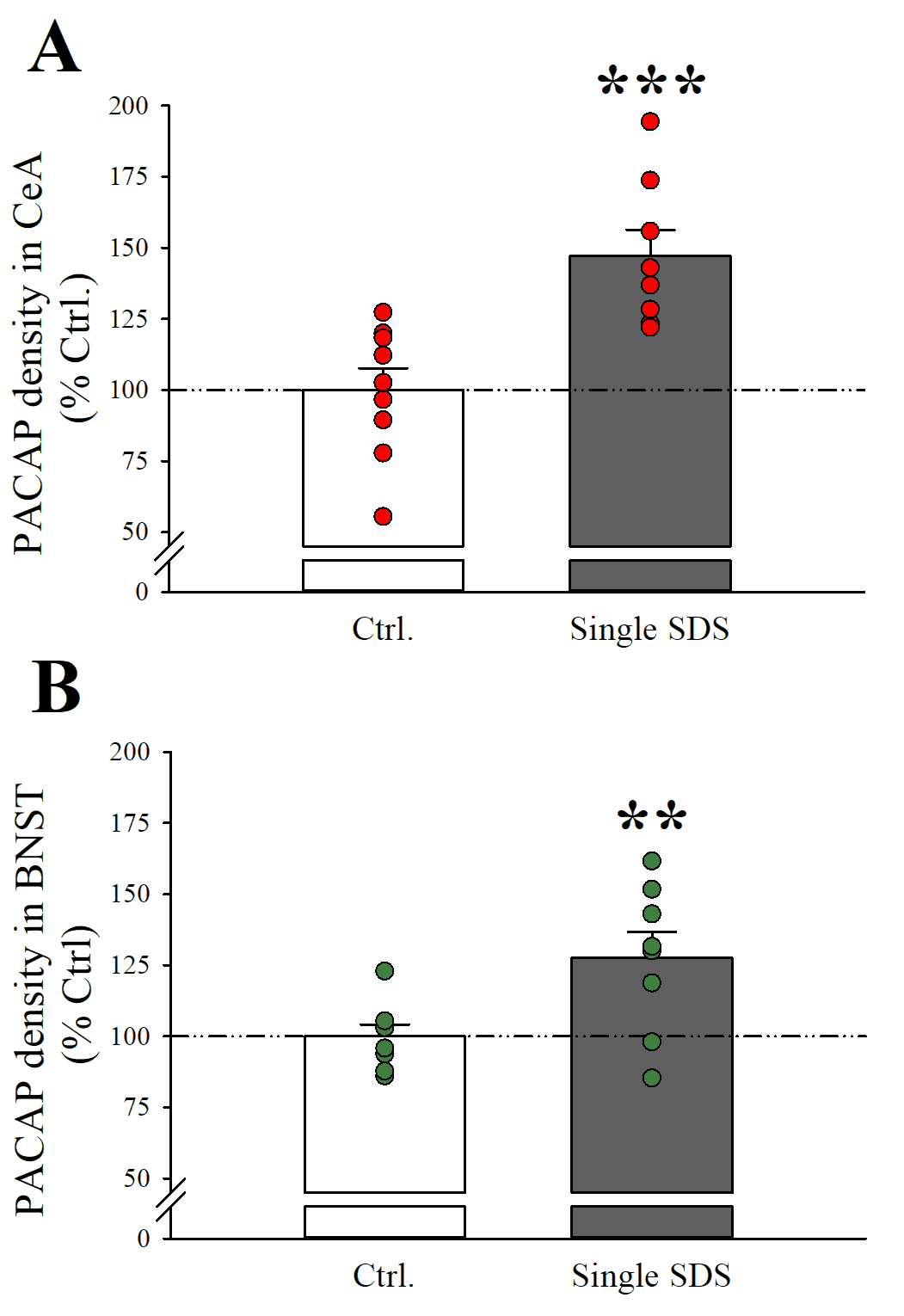

Supplement: Extended Data Figure 1-2 — Effects of 1 single social defeat session (Single SDS) on PACAP immunoreactivity in the CeA (A) and BNST (B) of rats. N= 8-9/group. Bars represent Mean ± SEM. ** p < 0.01, *** p < 0.001 vs. Ctrl. Download Figure 1-2, TIF file. [file enu-eN-NWR-0260-22-s03.tif]
